# Proteomic signature profiling in the cortex of dairy cattle unravels the physiology of brain aging

**DOI:** 10.3389/fnagi.2023.1277546

**Published:** 2023-12-07

**Authors:** Flora Cozzolino, Luisa Canè, Maria Claudia Gatto, Ilaria Iacobucci, Luigi Sacchettino, Davide De Biase, Evaristo Di Napoli, Orlando Paciello, Luigi Avallone, Maria Monti, Danila d’Angelo, Francesco Napolitano

**Affiliations:** ^1^CEINGE-Biotecnologie Avanzate “Franco Salvatore”-Via G. Salvatore, Naples, Italy; ^2^Department of Chemical Sciences, University of Naples, Naples, Italy; ^3^Department of Translational Medical Sciences, University of Naples Federico II, Naples, Italy; ^4^Department of Veterinary Medicine and Animal Production, University of Naples Federico II, Naples, Italy; ^5^Department of Pharmacy, University of Salerno, Fisciano, Italy

**Keywords:** bovine, brain, cortex, aging, proteomics

## Abstract

**Introduction:**

Aging is a physiological process occurring in all living organisms. It is characterized by a progressive deterioration of the physiological and cognitive functions of the organism, accompanied by a gradual impairment of mechanisms involved in the regulation of tissue and organ homeostasis, thus exacerbating the risk of developing pathologies, including cancer and neurodegenerative disorders.

**Methods:**

In the present work, for the first time, the influence of aging has been investigated in the brain cortex of the Podolica cattle breed, through LC–MS/MS-based differential proteomics and the bioinformatic analysis approach (data are available via ProteomeXchange with identifier PXD044108), with the aim of identifying potential aging or longevity markers, also associated with a specific lifestyle.

**Results and discussion:**

We found a significant down-regulation of proteins involved in cellular respiration, dendric spine development, synaptic vesicle transport, and myelination. On the other hand, together with a reduction of the neurofilament light chain, we observed an up-regulation of both GFAP and vimentin in the aged samples. In conclusion, our data pave the way for a better understanding of molecular mechanisms underlying brain aging in grazing cattle, which could allow strategies to be developed that are aimed at improving animal welfare and husbandry practices of dairy cattle from intensive livestock.

## 1 Introduction

Brain aging is an ongoing and unstoppable process characterized by physiological behavioral changes and cognitive decline, in terms of memory and executive function impairments (Blazer et al., 2015). In addition, morphological and biochemical changes, including deposition of pathological proteins, which eventually may turn into neuronal loss, can also occur in the nervous system network. In this line, dendrite remodeling, along with a decrease of axon number and synaptic plasticity disruption, are being observed in different brain regions of aged people and animals, including the neocortex (Burke and Barnes, 2006; Pannese, 2010; Dorszewska, 2013). In a previous behavioral and morphological study, a substantial reduction in dendritic spine number and thickness, as well as a poor performance on the delayed non-matching-to-sample test, was documented in the dorsolateral prefrontal cortex of non-human primates, thus highlighting a potential impact of the age-related axo-spinous synapses on cognitive functions (Dumitriu et al., 2010). Similarly, loss of synaptic density population and prefrontal cortex atrophy is generally associated with deficits in executive function, working memory, and increased perseveration in older healthy individuals. In this respect, in a recent work from Blinkouskaya and Weickenmeier (2021), the authors, taking advantage of large-scale crosssectional medical imaging studies, documented that aging is associated with a picture of progressive brain volume loss, cortical thinning, ventricular enlargement, and working memory deterioration in healthy humans (Blinkouskaya and Weickenmeier, 2021). A more detailed longitudinal analysis sought to correlate brain thickness, volume, and cognitive abilities with physiologic aging in middle-aged and older adults (Sele et al., 2021). In addition to imaging studies, electrophysiological evaluations confirmed the age-dependent loss of synaptic plasticity, as both elderly humans and animals displayed a similar altered form of long-term potentiation induction and maintenance (Morrison and Baxter, 2012; Arias-Cavieres et al., 2017; Temido-Ferreira et al., 2019; Di Lorenzo et al., 2020). The correlation between physiologic aging and extensive loss of synaptic connectivity might be overall prodromal symptoms for the development of neurodegenerative pathologies, such as mild cognitive impairment or Alzheimer’s disease (AD) in mammals (Harada et al., 2013; Masliah et al., 2013). Accordingly, cortical beta-amyloid (Aβ) deposition seems to affect up to 30% of elderly people with no neurodegeneration. Cortical atrophy might also be associated with the huge amount of misfolded Aβ protein deposits occurring during aging, accumulated in the extracellular milieu, which are thought to be associated with AD-like phenotypes in several domestic animals, including non-human primates, bovines, bears, domestic cats, and dogs. Together with cognitive alterations, several molecular pathways, such as oxidative stress, mitochondrial dynamics, autophagy, inflammation, and calcium homeostasis, are regarded as key factors affected by brain aging in most mammals (De Biase et al., 2017; Mattson and Arumugam, 2018; Drulis-Fajdasz et al., 2021; Zia et al., 2021). Accordingly, high-throughput proteomics identified several proteins of cognitive trajectory in human brains, belonging to mitochondrial activity, whose unbalance can lead to dysfunctions, observed early in AD onset and progression (Wingo et al., 2019). Likewise, the mass spectrometry-based technique revealed a significant reduction of the molecular machinery involved in the excitatory and inhibitory transmission in both hippocampus and cortex of 22-month-old C57BL/10 J mice (Drulis-Fajdasz et al., 2021). In the present study, by means of a large-scale proteomic approach devoted to the identification and relative quantification of proteins differentially expressed in the cortex of aged grazing Podolica cattle breed, we unraveled several biological processes encompassing synaptic plasticity, myelination, dendritic development, and oxidative stress, which were unbalanced or impaired when compared to the adult samples. Overall, the identification of potential aging or longevity markers might provide a greater understanding of the living conditions of cattle from intensive livestock farming, which often undergo early aging and eventually experience a detrimental impact on their welfare.

## 2 Results

### 2.1 Experimental workflow for protein identification and quantification

To comprehensively understand the biological processes that occur throughout the lifespan of healthy dairy cattle brains, identification of new potential biomarkers of brain aging was performed by employing a proteomics approach. Two cohorts of five dairy adult (7–12 years old) and five aged (16–24 years old) cattle were enrolled for this aim. The clustering of the samples was mainly in line with a previous work from Hoffman and Valencak, who reviewed the few published studies about the lifespan of domestic animals, including cattle (Hoffman and Valencak, 2020). In this respect, the authors reported that the maximum lifespan recorded for cattle is likely 20 years on average, depending on the breed, and described the only adulthood phase, after 12 years old. Therefore, given that the animals enrolled in the present study were far older (up to 24 years), we considered cattle from 7 to 12 years to be the adult group (since they are further from 20 years) and cattle from 16 to 24 years as aged, in order to make the sample size more homogeneous at the physiological level.

**Figure 1:**
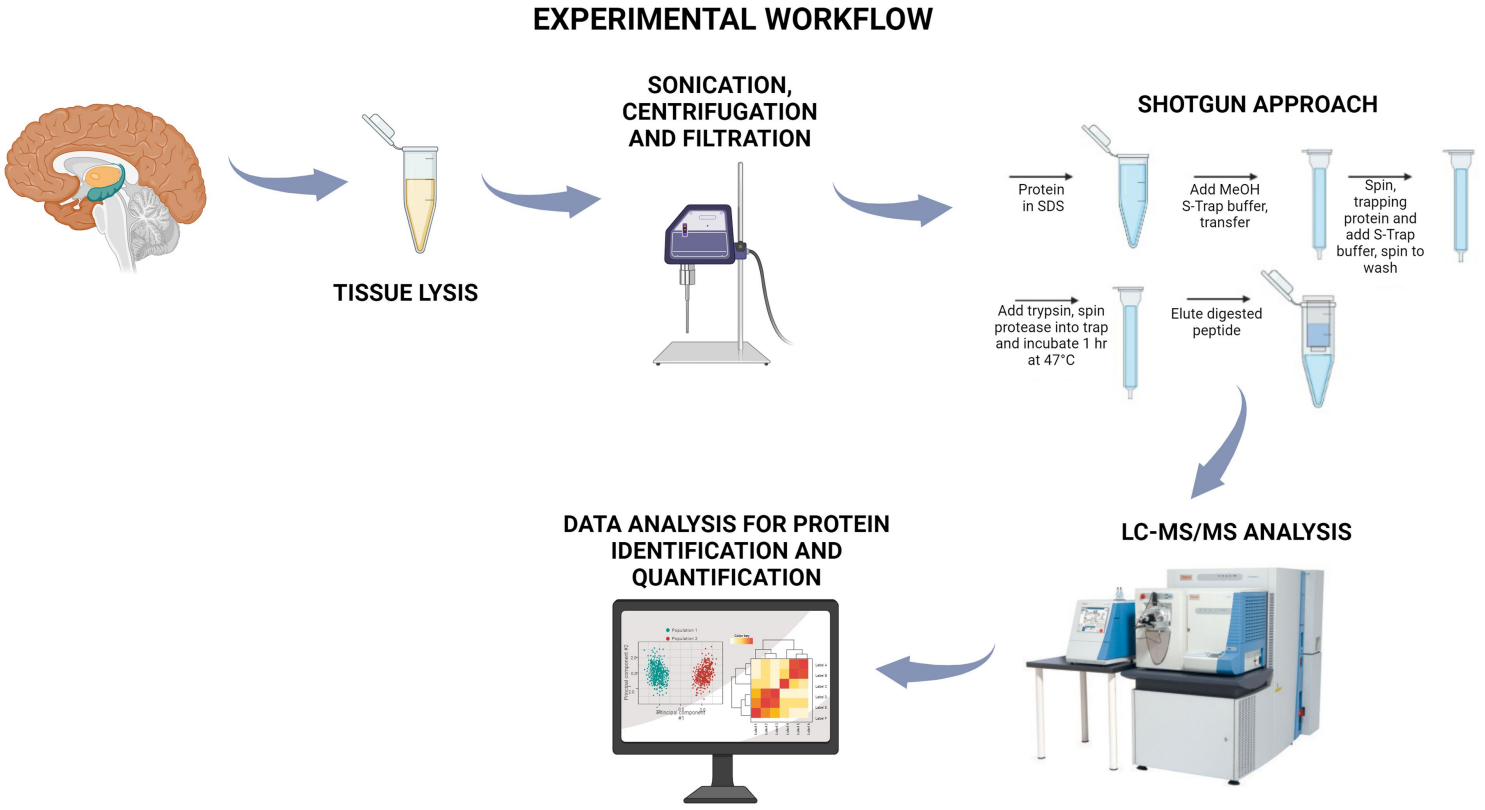
Workflow of the shotgun approach followed in the present study. Protein extracts derived from the cerebral cortex of adult and aged dairy cattle were subjected to a shotgun approach onto S-Trap filters, where, after the trapping, the samples were treated with trypsin to achieve protein digestion. The peptide mixtures were analyzed by nano LC–MS/MS, and proteins were identified and differentially quantified. Created with BioRender.com.

The cortex region was taken post-mortem and analyzed by a comparative label-free proteomics workflow, as reported in Figure 1. Proteins extracted from each cerebral cortex sample were subjected to a shotgun protocol relying on trypsin hydrolysis by using S-Trap cartridges (Iacobucci et al., 2022). Peptide mixtures were then analyzed by LC–MS/MS, and the raw data was processed by MaxQuant for protein identification and quantification.

**Figure 2:**
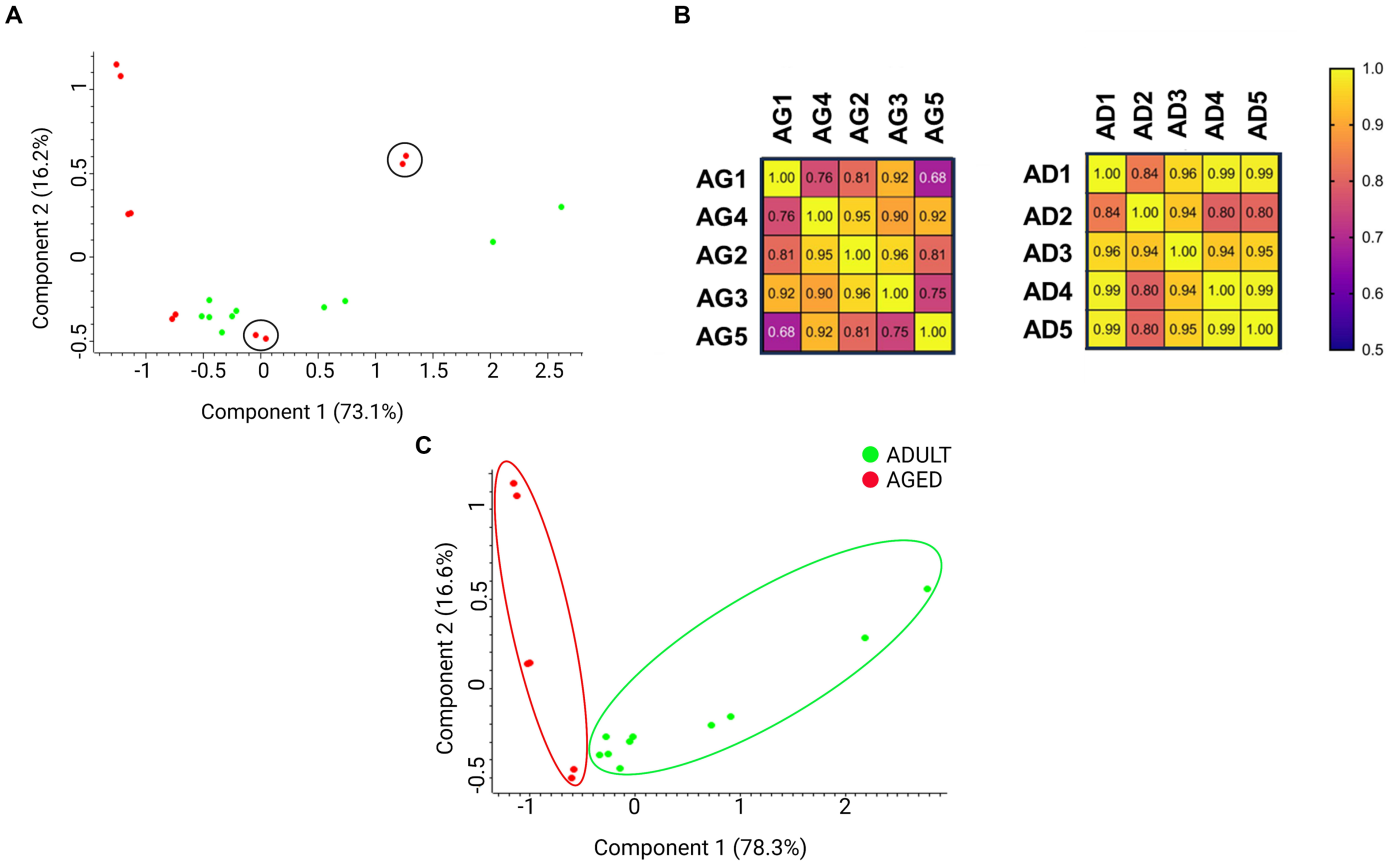
Principal component analysis (PCA) displays the distribution of biological samples from adult and aged cerebral cortex conditions on a two-dimensional graph. **(A)** Distribution of all samples in both aged and adult conditions. The aged 4 (AG4) and aged 5 (AG5) (in red and circled) samples were distant from the cluster containing all the other aged samples. **(B)** Correlation matrix based on Pearson coefficients. The aged and adult samples have been compared on the left and right matrix, respectively. **(C)** Distribution of samples without AG4 and AG5.

A preliminary principal component analysis (PCA) was carried out starting from the protein group generated by MaxQuant and containing 804 proteins, including contaminants, to display the distribution of biological samples from all studied conditions on a two-dimensional graph (Figure 2A). A PCA distribution can be considered appropriate when the biological samples belonging to the same condition are well grouped in a cluster and, contemporaneously, they are well separated from the other groups under examination. As shown in Figure 2A, the aged samples 4 (AG4) and 5 (AG5) (red dots in black circles) were very distant from the other aged samples (dots in red in Figure 2A), suggesting a high level of heterogeneity from a biological point of view. To further support the hypothesis, we drew a correlation matrix based on the Pearson coefficient (Figure 2B). As displayed in the left panel of Figure 2B, the two samples showing a lower homogeneity when compared to the other of the same group were again AG4 and AG5 (Pearson coefficient < 0.8). This results confirms the PCA plot results. Conversely, all adult samples show correlation index ≥0.8 (Figure 2B, right panel), suggesting a high correlation level among all of them. Therefore, we decided to select the most homogeneous samples for the following analyses. MaxQuant processing was repeated excluding AG4 and AG5 samples; the new outcome of the PCA plot is shown in Figure 2C. The new protein group, containing 776 proteins (Supplementary Table S1), was then imported into Perseus for normalization, imputation, and subsequent statistical analysis (see methods section).

Fold changes (FCs) of statistically significant proteins were calculated as the ratio of LFQ intensities measured for each protein in aged vs. adult (control) samples. We assumed biologically significant log_2_FCs > 0.5 for up-regulated and log_2_FCs < −0.5 for down-regulated proteins. The lists of the up- and down-regulated proteins, including the FCs values, are reported in Supplementary Table S1.

The largest number of identified proteins was down-regulated (88), while less than half (42) were found to be up-regulated. These numbers suggested that a prevalent loss of functions occurs in the aged brain cortex in comparison with adults.

**Figure 3:**
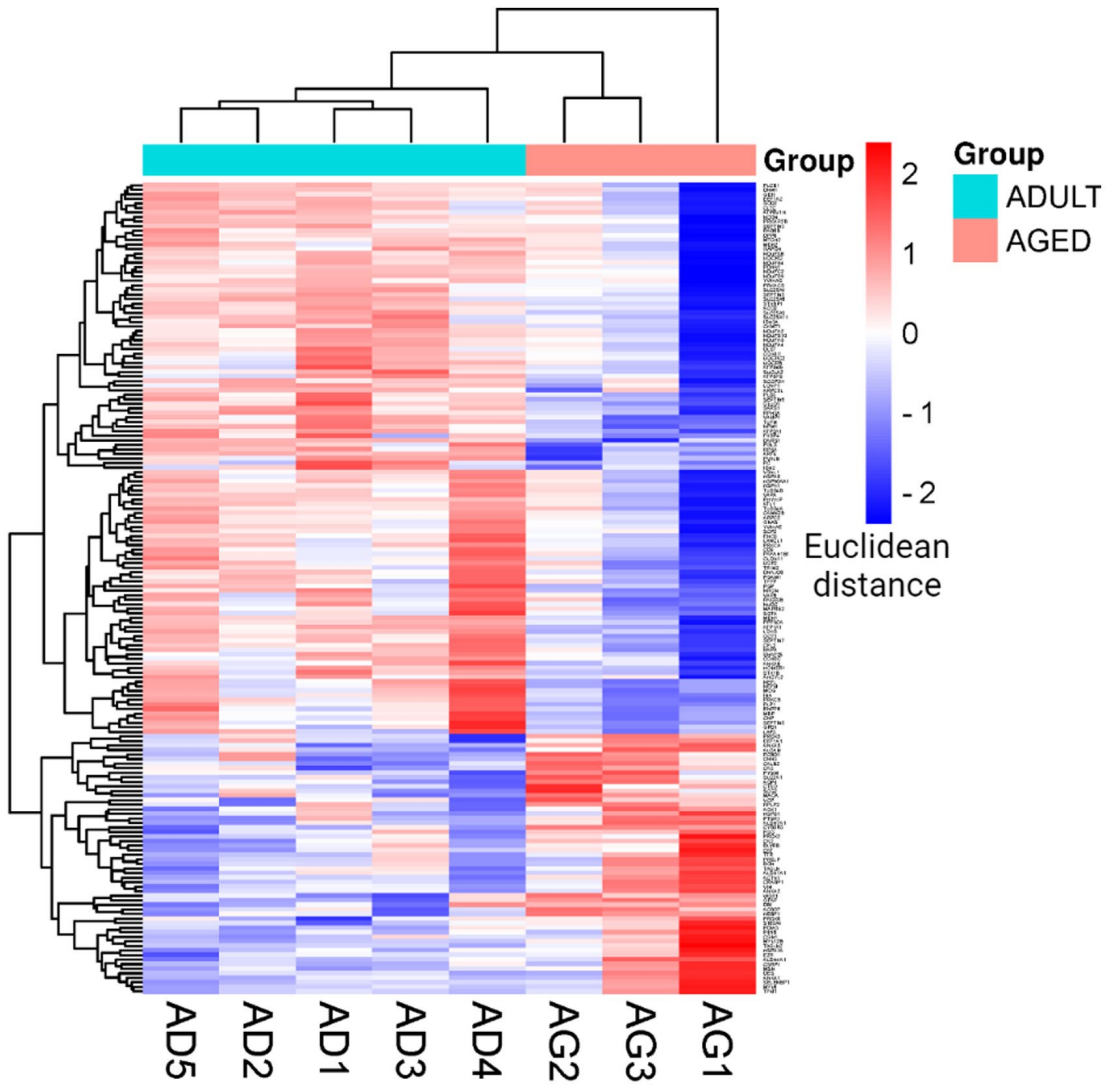
Cluster heatmap. Heatmap was plotted by https://www.bioinformatics.com.cn/en, a free online platform for data analysis and visualization. Each row represents individual genes, corresponding to up- and down-regulated proteins in the adult and aged cerebral cortex, and each column indicates a sample analyzed for aged (AG): AG1, AG2, AG3; and adult (AD): AD1, AD2, AD3, AD4, AD5.

To better visualize the expression profiles in all samples, a hierarchical clustering analysis was performed on the set of statistically significant proteins according to the LFQ intensities measured for each protein in each sample. This method generates a visual heatmap (Figure 3) that allows monitoring of the levels of protein expression across all the samples, reporting in green the proteins showing lower expression levels and in red those showing higher expression levels. The rows are clustered and arranged according to their heat map response, and a dendrogram is provided to display the similarity of protein expression (Figure 3).

### 2.2 Functional analysis

**Figure 4:**
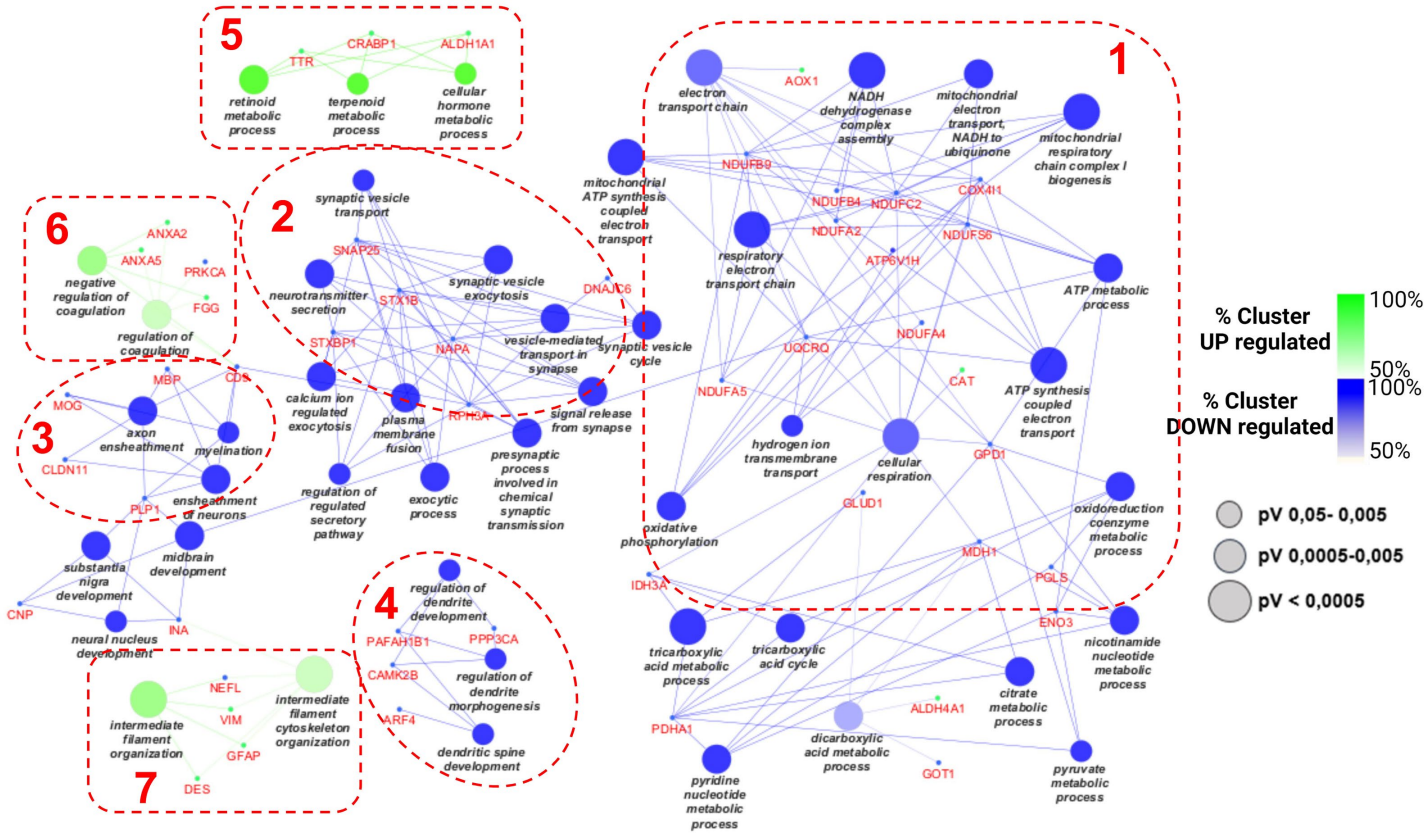
Cytoscape graphical representation of cellular processes involving up- (green) and down-regulated (blue) proteins. The GO database was used; a value of *p* Benjamini-Hochberg correction cutoff of 0.05 was applied. Node sizes were correlated with the specific value of ps according to the legend reported in the picture.

The lists of 42 up- and 88 down-regulated proteins were analyzed by Cytoscape software, with the ClueGO app + CluPEDIA extension, to visualize the protein networks that were up- and down-regulated in the aged condition in comparison with the adult protein set. The analysis was performed using Biological Process as the setting and Benjamini-Hochberg as the Statistical Options, with a cutoff of FDR ≤ 0.05. The resulting Cytoscape networks are reported in Figure 4.

As expected, based on the different numbers of up- and down-regulated proteins, the largest part of proteins merged into down-regulated processes, as reported in blue. The green clusters refer to the up-regulated pathways. Four considerably populated down-regulated clusters were visualized. The largest one (on the right side of the picture, cluster 1) refers to the impairment of some mitochondrial activities, such as electron transfer chain and ATP synthesis, as well as the tricarboxylic acid metabolic process. Many subunits of NADH dehydrogenase complex are present (NDUFB4, NDUFB9, NDUFA2), as well as many other mitochondrial enzymes (IDH3A, GLUD1, COX4I1, GPD1). The second pathway in terms of gene enrichment (cluster 2 in Figure 4) concerned synaptic vesicle transport and includes DNAJC6, SNAP25, STX1B, STXBP1, and NAPA.

The other two down-regulated processes refer to myelination and dendritic spine development (clusters 3 and 4 in Figure 4, respectively) and include PLP1, MBP, MOG, CNP, INA, and PP3CA; and CAMK2B and PAFAH1B1, respectively. Only a few proteins were associated with each of the three up-regulated processes: TTR, CRABP1, and ALDH1A1 for the “cellular hormone metabolic process” (on the top of the picture, cluster 5 in Figure 4); ANXA5, ANXA2, PRKCA, and FGG for the so-called “regulation of coagulation” process (cluster 6 in Figure 4); and NEFL, VIM, GFAP, and DES for the “intermediate filament cytoskeleton organization” (cluster 7 in Figure 4).

**Figure 5:**
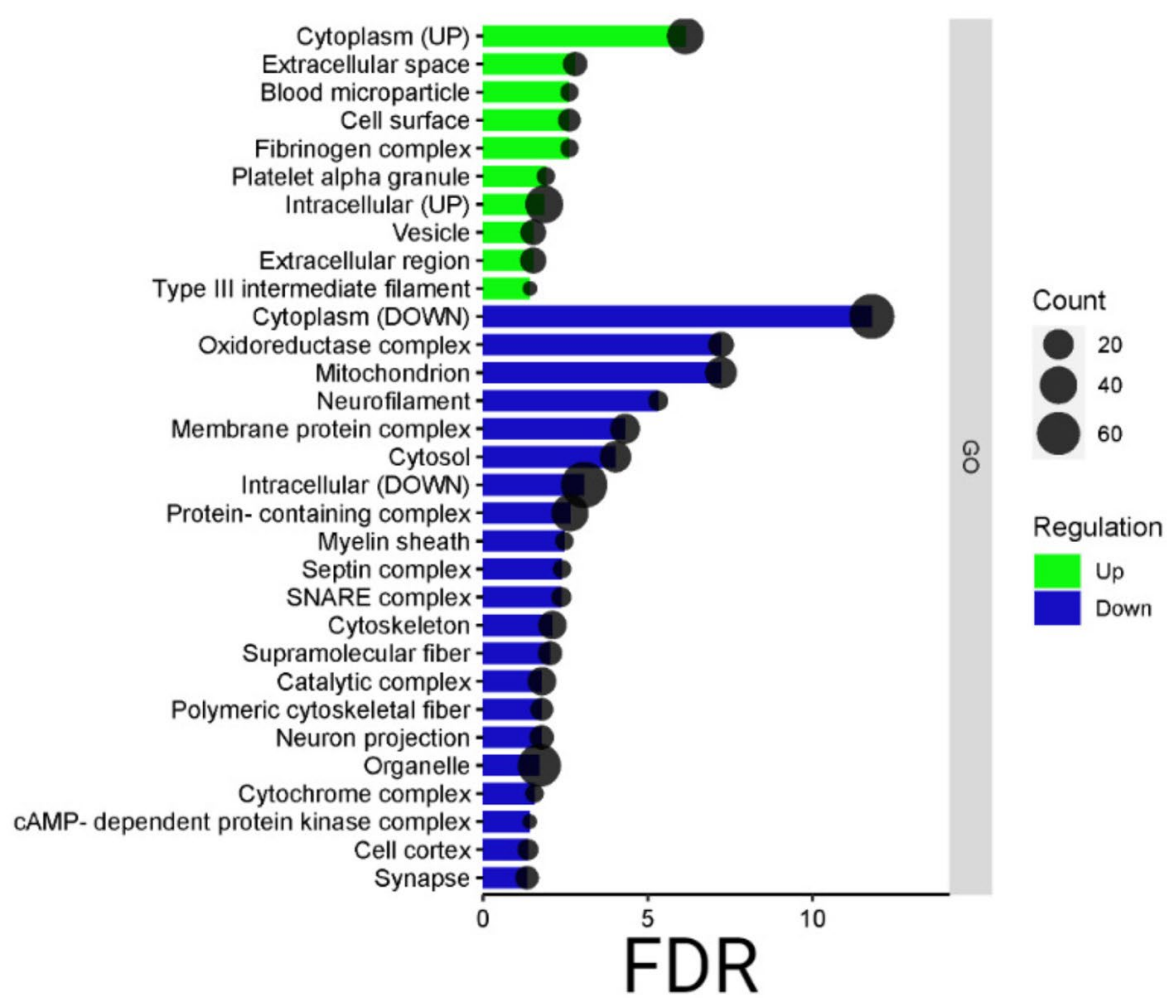
Bardot subcellular component. Bar dot graphical representation of STRING subcellular component: count is the number of proteins grouped in the GO term, in green up-regulated proteins, and blue down-regulated ones. On the *y*-axis, the subcellular component terms are reported, while on the x-axis the false discovery rate (FDR).

In addition, the STRING analysis, reported in Figure 5, concerning the distribution of up- and down-regulated proteins among subcellular components, is in line with the Cytoscape results. Indeed, the highest part of down-regulated proteins is resident in cytoplasm, mitochondria, and neurofilament, while the up-regulated proteins are distributed among the cytoplasm, extracellular space, and vesicle.

### 2.3 Protein fold changes validation

A pool of proteins belonging to the main functional clusters was selected for validation experiments, relying on an innovative antibody-free approach and based on LC–MSMS methodologies, known as multiple reaction monitoring (MRM). By using predictive software, as well as Skyline, proteotypic peptides were selected for each protein target, and MRM experiments were set up to monitor specific transitions, defined by the m/z of parent ion and the m/z of daughter ions generated from the fragmentation procedure, according to each peptide amino acid sequence. Tryptic mixtures prepared from all adult and aged samples were separated by nano LC–MS/MS in MRM mode, and two or more transitions of at least three proteotypic peptides were monitored for each protein. Each sample was run in duplicate, and the areas recorded for each transition were mediated among technical replicates, with all peptides belonging to the same protein. The final average values, normalized with the areas measured for ACTIN-derived transitions in the same sample, were statistically analyzed and employed to calculate the FCs. Table S2 summarizes the proteins validated with the relative peptides, transitions, MRM data, and the FCs measured by this targeted approach in comparison with the untargeted method. Analytical data were processed by GraphPad Prism 9. The results were depicted in a two-dimensional graph, where the intensity of the normalization area for each analyzed protein is indicated.

**Figure 6:**
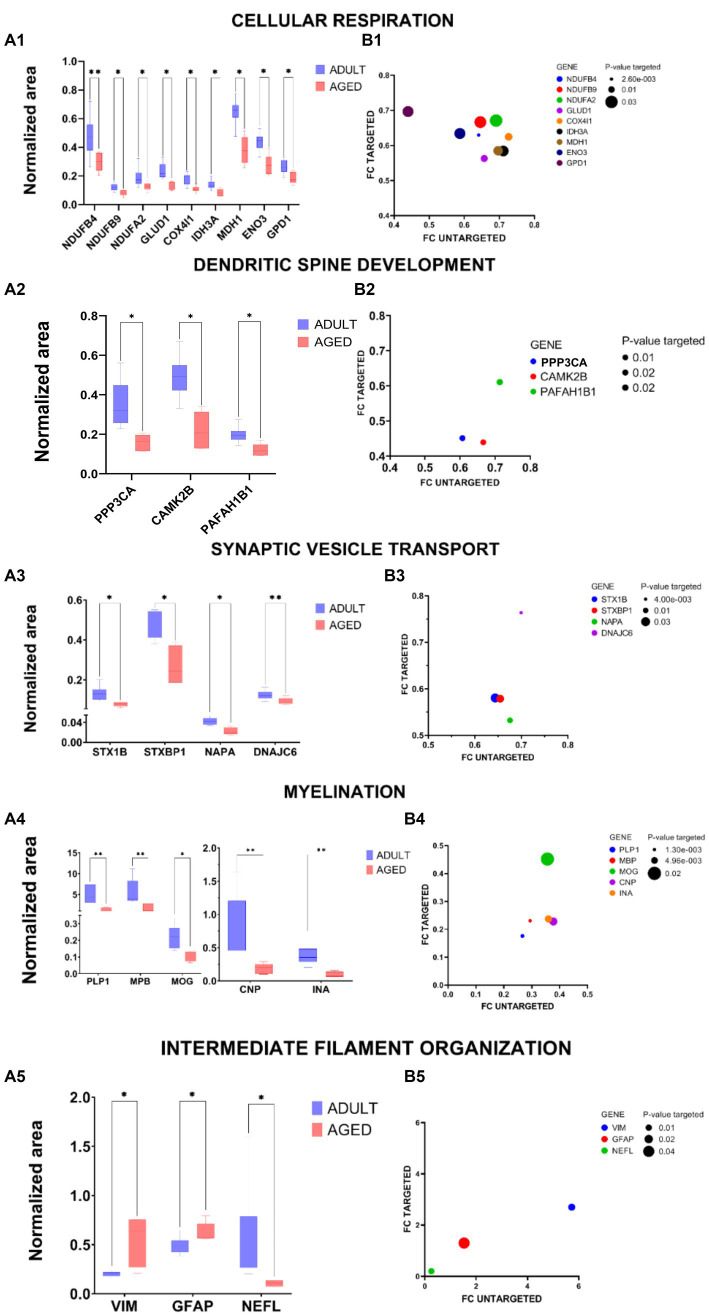
Graphical representation of MRM experiments. Panels A: the normalized area of selected proteins measured in aged vs. adult samples for specific proteins selected within cellular Respiration **(A1)**, dendritic spine development **(A2)**, synaptic vescicle transport **(A3)**, myelination **(A4)**, and intermediate filament organization **(A5)** are reported in two-dimensional graphs; the statistical significance of protein expression calculated as aged/adult with a value of *p* <0.05 (*) and value of *p* <0.01 (**) are also indicated. Panels B: correlation graphs of FCs of validated proteins. The FCs measured in the MRM experiments (FCs targeted) for specific proteins selected within cellular respiration **(B1)**, dendritic spine development **(B2)**, synaptic vescicle transport **(B3)**, myelination **(B4)**, and intermediate filament organization **(B5)** are reported in function of FCs measured in the untargeted approach, according to LFQ intensities. The ball dimensions respect the *p*-values of targeted validations. The correlation graphs show the consistency of the FCs measured in the target method (MRM experiments) with those obtained in the label-free untargeted approach.

In all “A” panels of Figure 6, the validation of proteins with *p* < 0.05 were reported, and for all validated proteins, a correlation graph (“B” panels) relative to the FCs measured in targeted and untargeted approaches is also reported. Some proteins, one belonging to the “cellular hormone metabolic process” (TTR) and one to the “regulation of coagulation” (ANXA2), were also monitored by the MRM method, but although their trends were in agreement with those detected in the untargeted approach, their quantification did not satisfy the quality thresholds applied, either in terms of numbers of peptide detected or in terms of statistical significance (see Supplementary Table S2). In some cases, such as TTR, it was most likely due to the small number of proteotypic peptides available for each protein. Despite these few exceptions, as previously reported (Cozzolino et al., 2020), MRM methodology not only resulted in being very powerful in the validation procedure—able to be independent because of the purchase of many antibodies, not always commercially available—but, additionally, the quantitative data (FCs) were very robust, as demonstrated by the fact that they are largely in agreement with those produced by the label-free quantification in the untargeted approach.

## 3 Discussion

Physiologic brain aging relies on functional and structural time-dependent changes that lead to a reduction of both homeostasis and functional abilities. Indeed, a general decline in overall cerebral volume, associated with ventricular enlargement, white matter hyperintensities, and alterations of its microstructural properties, occurs in mammalians over time. A decrease in brain membrane composition in aged samples, from 40 years of age and for each future decade throughout the lifespan of humans, has accordingly been documented (Svennerholm et al., 1997; Scahill et al., 2003; Peters, 2006). Age-dependent changes in brain volume and composition, caused by loss of dendritic spines and physiological neuroplasticity, bring about alterations in complex brain circuitries overseeing cognitive skills, including executive functions and implementation of goal-directed behavior, as assessed in rodents, non-human primates, and humans (Rapp and Amaral, 1989; Morrison and Baxter, 2012). In the present work, we evaluated the potential impact of physiologic aging upon proteomic profiling in the cortex of grazing Podolica cattle breed and identified 130 differentially expressed proteins. In particular, the protein–protein interaction network, and further validations by the MRM approach, showed that pathways were associated with cellular respiration, dendritic spine development, synaptic vesicle trafficking, myelination process, and intermediate filament organization, and were significantly down-regulated in the aged animals, when compared to the adult controls.

### 3.1 Cellular respiration

Most of the downstream cortex-dependent events associated with higher cognitive functions require an intact mitochondria homeostasis that, when impaired, might be causative of age-related metabolic and behavioral dysfunctions. Mitochondria are subcellular self-autonomous organelles responsible for ATP production (through oxidative phosphorylation), supporting almost all cellular processes, including regulation of intracellular calcium homeostasis, neurotransmitter synthesis, and synaptic plasticity, as well as lipid metabolism and regulation of apoptosis. Our data showed a significant alteration of the activity in the electron transport chain complex I (NADH–ubiquinone oxidoreductase) since NDUFA2, NDUFB4, and NDUFB9 protein levels were down-regulated in the cortex of the old samples. Its decreased activity turns out to be in slower NADH degradation rates, which can affect both anaerobic and aerobic energy metabolism. As a result, the overall decrease in ATP production deprives brain cells of the energy they need to perform their physiological functions (Mohamad Kamal et al., 2020). Moreover, we found that proteins belonging to tricarboxylic acid/dicarboxylic acid metabolic processes, namely glutamate dehydrogenase, isocitrate dehydrogenase, and malate dehydrogenase 1 (Dong and Brewer, 2019; Aldana et al., 2020; Bayliak et al., 2022), were down-regulated in the old animal samples, thus allowing us to hypothesize a crucial role of the mitochondrial homeostasis and metabolism upon the modulation of the age-dependent bioenergetics and cognitive processes.

### 3.2 Dendritic spine development

In this respect, cognitive impairments during physiologic aging can also be related to the deterioration of neuronal branching, which basically consists of dendritic spines, and represents postsynaptic sites of most excitatory synapses in the mammalian brain. Dendritic spines deletion was found in both the sensory and motor cortex of old mice, when compared to young and adult animals, through high-resolution two-photon imaging studies. Moreover, spine-density changes over time correlated with behavioral functions, as old animals showed compromised learning abilities, so they were less responsive to modulation by learning experiences, likely because a deterioration of synaptic function and cognitive abilities occurred (Huang et al., 2020). Proteomics data showed that both calcium/calmodulin and calcineurin levels were significantly reduced in the old cortex samples. Calcium/calmodulin-dependent protein kinase II (CaMKII) is a ubiquitous and central component of Ca^2+^ signaling, best known for its functions in the brain, and it has been extensively studied, particularly in the context of dendrite development and synaptic long-term potentiation (Nicole and Pacary, 2020). The α isoform of CaMKII has been extensively studied and considered a key modulator of neuronal plasticity. In contrast, out of the remaining three CaMKII isoforms (β,γ,δ), little attention was paid to CaMKIIβ in brain physiology until not too long ago. Together with CaMKIIα, highly expressed in the brain, the β isoform was essentially detected in the cerebellum, hippocampus, and cortex, regions associated, among others, with cognition, motor coordination, and mood-related behavior, respectively. Indeed, the lack of CAMKIIβ caused ataxia and cognitive deficits, with a reduction of anxiety-related behavior in CAMKIIβ knockout mice (van Woerden et al., 2009; Bachstetter et al., 2014). CaMKIIβ may have multiple functions in the final stages of the neuronal development process, particularly during neuronal migration, dendrite morphogenesis, and backbone/synapse formation (Nourbakhsh and Yadav, 2021). Moreover, disruption of CaMKIIβ expression has been linked to neurodevelopmental and synaptic plasticity alterations, and Cho et al. (2007) documented that transgenic mice, which expressed an elevated CaMKIIβ activity, displayed reduced LTP in the perforant path of the dentate gyrus (Cho et al., 2007), thus suggesting its potential impact on brain diseases. The 61-kDa calmodulin-binding catalytic subunit A is a serine/threonine phosphatase encoded by the PPP3CA gene, which controls the phosphorylation states of both excitatory and inhibitory receptors. Among wide physiological activities, PPP3CA influences synaptic transmission through calcium-dependent modulation onto excitatory postsynaptic dendritic spines in neurons of several brain regions, such as hypothalamus Purkinje cells of the cerebellum and cortex (Tarasova et al., 2018).

### 3.3 Synaptic vesicle transport

The functional role of the platelet-activating factor acetylhydrolase 1B1 (PAFAH1B1) is related to cortical development, and previous studies documented that genetically modified mice with Pafah1b1 haploinsufficiency displayed defects in neuronal migration, cognitive deficits, epilepsy, and disorganization of some brain areas, including cortex and hippocampus, most likely associated with selective reductions of cortical GABAergic interneurons (Hirotsune et al., 1998; Paylor et al., 1999; Pleasure et al., 2000; Greenwood et al., 2009; Dinday et al., 2017). At the synaptic level, mechanisms by which vesicles release neurotransmitters that, eventually, deliver signals from one cell to another are thought to be relevant in cognitive processes. Age-dependent alterations in synaptic vesicle release machinery might bring about severe implications upon the transfer of information to postsynaptic neurons within brain regions associated with executive functions, as well as learning and memory abilities. Our data showed a significant impairment of cortical SNARE complex assembly, as the levels of syntaxin-binding protein 1, alpha-soluble NSF attachment protein (NAPA) were significantly lower in the aged cattle when compared to the adult animals, thus suggesting the importance of both presynaptic and postsynaptic machinery in modulating cognitive functions in bovine.

### 3.4 Myelination

The electrical insulator myelin is organized as a multilamellar membrane so that axons are allowed to be protected and make saltatory conduction more efficient (Simons and Nave, 2015). Although the functional role of aging on the expression of myelin proteins is not yet fully understood, the well-known decrease of the myelin-related markers, such as myelin basic protein and proteolipid protein, at both transcriptional and translational levels, is widely regarded as one of the key factors underlying age-dependent alterations in neuronal networking and cognitive abilities in animal models and humans as well (Xie et al., 2013; Chapman and Hill, 2020; Rivera et al., 2021). A gradual oligodendrocyte death, associated with myelin debris accumulation, was described in the cortex of 1-year-old creER transgenic mice, thus strengthening the hypothesis of a crucial role played by myelin in synaptic plasticity homeostasis (Hill et al., 2018).

### 3.5 Intermediate filament organization

Alongside a crucial involvement of mitochondrial dysfunction in cellular aging processes, proteins underpinning cytoskeleton remodeling processes are being regarded as senescence markers at both peripheral and central nervous system levels (Kim et al., 2022). In this respect, intermediate filament proteins are known to provide support and structure for cells, as well as to modulate mitochondrial motility (Tolstonog et al., 2005; Schwarz and Leube, 2016), which can be generally subjected to changes in response to alterations in energy demand and nutrient availability, namely aging and age-related disorders (Chistiakov et al., 2014). In this respect, stereological and morphological studies in human and mouse models documented an age-dependent increase of vimentin and GFAP proteins in the astrocytes of different brain regions, including hippocampus, prefrontal cortex, thalamus, and caudate nucleus (Salminen et al., 2011; O’Leary et al., 2020). Our data showed a higher increase of glial fibrillary acidic protein (G-FAP), associated with a significant down-regulation of the neurofilament light chain protein (NFEL), in the cortex of the old samples when compared to the younger ones, thus suggesting mostly common age-dependent changes across species.

Brain aging is a complex and multifactorial physiological process, induced by genetic, epigenetic, and environmental factors, which could affect neurons and cause mammals to develop behavioral disorders, dementia, and impaired immune response over time (Costantini et al., 2018; Abdalla et al., 2022). In this line, previous studies in the same breed of cattle performed by Paciello and colleagues showed that, in line with other animal species, aged bovine showed a build-up of lipofuscin and a progressive reduction of autophagy initiation, as APP protein levels were higher in both hippocampus and dentate gyrus of old animals, associated with lower levels of Beclin-1 and higher expression of LC3 when compared to the young control brain samples (De Biase et al., 2017). Alongside encouraging and novel data, some relevant concerns and experimental limitations of our study should be taken into account and thoughtfully addressed. The Podolica cattle breed enrolled had no parental bond, as they came from several different extensive family-run farms in Salerno province, in Southern Italy. They had the freedom to wander outdoors and autonomy over access to shelter, water consumption, and diet selection, mostly by grazing on large areas of pastures and natural resources. Moreover, the animals were held in farms that offered very good welfare conditions, such as protection from predators and extreme changes of temperature, which allowed the animals to form natural bonds and hierarchies and to be free from thirst, hunger, and fear. Therefore, animal welfare was not negatively influenced by intense noises, human activities, inadequate husbandry management, or poor air quality. In addition, we did not find any gross neurological or physical abnormalities in the cattle enrolled in our study. However, considering that behavioral studies were not performed on these animals, we cannot draw any potential correlation between cortical-dependent alterations at the proteomic level and cognitive impairments, if any. It should be pointed out that we only acquired samples after animals were taken to the slaughterhouse following their natural death. In addition, it should be noted that the Podolica cattle breed are not as widespread as livestock farming animals, who are slow growing and expensive to raise. In our experimental conditions, we did not observe any alterations either in autophagy or inflammation processes, most likely because of the experimental strategy employed. Indeed, the untargeted proteomic strategy that we used can substantially give a global overview of the whole proteome by facilitating the detection of the most representative pathways modulated within the cell or tissue over time. In general, all the omics sciences are high-throughput approaches, may be not able to identifiy, or underestimate, the differential expression of specific targets, which would rather require different experimental settings to be identified. Overall, our unbiased and proof-of-concept proteomic work highlights the importance of further characterizing and validating cortical age-dependent biomarkers, aimed at improving animal welfare and husbandry practices of dairy cattle from intensive farming livestock.

## 4 Materials and methods

### 4.1 Animals

For the present study, samples were collected from 10 dairy cattle (7–24 years old) in an abattoir in Campania Region, Italy, during postmortem inspection. Permission to obtain the samples was granted from the owner of the abattoir and from the veterinary inspector responsible for sanitary surveillance. Each animal underwent a physical examination, which did not report any apparent clinical illness or neurological signs (gait abnormalities, weakness, and decreased mental status). Afterward, the animals were slaughtered in strict accordance with European slaughter regulations (CE n° 1099/2009). Moreover, the absence of prior diseases was confirmed in all animals by performing the rapid test recommended by European law. Animals were divided into two groups, namely Group A (adult): 7–12 years (*n* = 5), and Group B (aged): 16–24 years (*n* = 5).

### 4.2 Protein sample preparation for shotgun proteomics analysis

Cerebral cortex samples from five adult and five aged dairy cattle were treated with lysis buffer (SDS) 5% (Sigma-Aldrich) and ammonium bicarbonate 50 mM (BioRad), and were subjected to 3 cycles of high-frequency sound wave sonication via a probe inserted in the sample, which creates an area of low pressure to favor the disruption of tissue. Following incubation at 4°C for 45 min on a bench spinning shaker, samples were centrifugated (30 min at 13,000 rpm). Supernatants were collected, and protein extracts were quantified by bicinchoninic acid assay (Thermo Fisher Scientific). Of each protein sample, 50 μg was digested by trypsin onto S-Trap filters according to the manufacturer’s protocol (ProtiFi, Huntington, NY) (Palinski et al., 2021). Peptide mixtures were dried in a SpeedVac system (Thermo Fisher Scientific).

### 4.3 LC–MS/MS analyses and protein identification and quantification

Peptide mixtures were dissolved in 60 μL of formic acid 0.2%, and 2 μL were analyzed on an LTQ Orbitrap XL (Thermo Fisher Scientific) coupled to the nanoACQUITY UPLC system (Waters). Samples were firstly concentrated onto a C18 capillary reverse-phase pre-column (20 mm, 180 μm, 5 μm, Waters Bioseparation Technology) and then fractionated onto a C18 capillary reverse-phase analytical column (250 mm, 75 μm, 1.8 μm, Waters Bioseparation Technology) working at a flow rate of 300 nL/min. A linear chromatographic gradient was used, consisting of a ramp of eluent B (0.2% formic acid in 95% acetonitrile) in eluent A [0.2% formic acid in 2% acetonitrile in liquid chromatography-mass spectrometry (LC–MS) grade (Merck)] from 5 to 95% in 201 min. Tandem mass spectrometric (MS/MS) analyses were performed in data-dependent acquisition (DDA) mode, by fragmenting the 10 most intense ions in the collision-induced dissociation (CID) method. All samples were run in technical duplicates (Andolfo et al., 2023). The software MaxQuant (v. 1.5.2.8), was utilized for the protein identification and quantification through raw files using the following parameters: UniProt Database; Taxonomy: *Bos taurus* (Bovine); enzyme: trypsin; maximum missed cleavages: 1; fixed changes: carbamidomethyl I; variable changes: oxidation (M); Gln-pyro-Glu (N-Term Q); minimum number of peptides for identification: 4 with at least 2 unique; minimum number of peptides for quantification 4 FDR: 0.01; min Score 10; and peptide tolerance 20 ppm.

### 4.4 Functional enrichment analysis

Differentially expressed proteins identified in the set of analyzed cortex samples were collected in a unique list, which was analyzed using the ClueGO + CluPEDIA extension app of Cytoscape (v3.9.1). The biological processes database of Gene Ontology (GO) *Bos Taurus* was queried by applying the Benjamini-Hochberg correction with a value of *p* <0.05. The bar-dot graph was plotted by https://www.bioinformatics.com.cn/en, a free online platform for data analysis and visualization, by using the STRING output based on the GO cellular component database.

### 4.5 Multiple-reaction monitoring analysis

Multiple-reaction monitoring (MRM) analysis was carried out for the validation of some biomarkers of each functional network. In particular, 50 μg of protein extracts obtained from different samples were digested by trypsin onto S-Trap filters, as described above.

By using Skyline v20.1.0.155 (MacCoss Lab Software, Dept of Genome Sciences, UW) for each protein, at least three prototypic peptides and at least three transitions for each parent ion were selected and monitored by using a Xevo-TQS triple-quad mass spectrometer, coupled to a nanoAcquity UHPLC (Waters, Milford, MA, US) equipped with IonKey CHIP interface. Peptide mixtures were separated on peptide BEH C18 130 A°, 1.7 μm, 150 μm x 50 mI, iKey by using a linear gradient of eluent B (95% acetonitrile LC–MS grade (Sigma-Aldrich), 0.2% formic acid (Sigma-Aldrich)) from 7 to 95% over 60 min working at a flow rate of 3 μL/min. Each run was analyzed in duplicate, and the total area of each peptide transition was used for the relative quantification of the specific proteins by using actin peptide ion transitions for normalization (Berkman et al., 2019).

### 4.6 Statistical analysis

The Perseus Software (1.6.15.0), used for statistical analysis of differentially expressed proteins, analyzed LFQ intensity values in accordance with its normalization and imputation criteria to produce a relative protein quantification. Proteins with more than 70% of non-valid values (zeros) were removed, and the data were normalized by applying a logarithmic scale [log_2_ (LFQ intensity)]. Additionally, imputation was used to allow the replacement of invalid values with numerical values that followed the Perseus mode (downshift of the standard deviation of 1.8 and a width of 0.3). The statistical significance of the difference in protein expression was determined using Student’s t-test. The distribution of the samples was shown using the platform option for principal component analysis (PCA) performed by Perseus software. For MRM experiments, the data of the selected proteins were analyzed using GraphPad Prism 9, employing a t-test to compare the data between the aged and adult conditions. The results were depicted in a two-dimensional graph, where the intensity of the normalization area for each analyzed protein was indicated, highlighting the significance of protein expression calculated as aged/adult with a value of *p* <0.05.

## 5 Conclusion

In conclusion, we identified, for the first time, 130 proteins differentially expressed in the cerebral cortex of the old bovine and associated with the decline of physiological brain functions. Proteomics and bioinformatic analysis revealed that aging is associated with the down-regulation of molecules involved in synaptic plasticity, myelination, dendritic process, and oxidative stress. Depending on the breed, dairy cattle lifespan may be up to 30 years. However, when milk productivity is lowered, it becomes about 6 years on average (Hoffman and Valencak, 2020). Longevity in livestock farms represents one of the main challenges to overcome and requires consideration of a combination of many different factors during the lifespan of a cow, ranging from the economic performance of farms to the welfare status of the animals (Dallago et al., 2021). Therefore, the present work could provide a basis for further and wider studies about proteomic profiling in dairy cattle from intensive livestock farming to try and identify potential aging markers in young animals and implement functional strategies to improve their quality of life and productivity.

## Data availability statement

The original contributions presented in the study are publicly available. This data can be found here: ProteomeXchange, PXD044108.

## Ethical statement

The study did not require consent or ethical approval according to European Directive 2010/63/EU because all sampling procedures from animals were performed during post-mortem inspection. However, the animals were slaughtered rigorously in line with European regulations (CE no: 1099/2009 of 24 September 2009) that assure the protection and welfare of animals at the time of killing. The owner of the abattoir and the veterinary inspector responsible for sanitary surveillance granted permission to collect the samples.

## Author contributions

FC: Investigation, Writing – original draft. LC: Methodology, Validation, Writing – review & editing. MG: Methodology, Validation, Writing – review & editing. II: Writing – review & editing, Data curation, Formal analysis, Software. LS: Writing – original draft. DD: Resources, Visualization, Writing – review & editing. ED: Resources, Visualization, riting – review & editing. OP: Resources, Visualization, riting – review & editing. LA: Project administration, Visualization, Writing – review & editing. MM: Conceptualization, Resources, Supervision, Writing – original draft, Writing – review & editing. Dd’A: Conceptualization, Writing – review & editing. FN: Conceptualization, Writing – review & editing, Project administration, Writing – original draft.
